# Supplementary material for: Targeting Immunosuppressive Tumor-Associated Macrophages Using Innate T Cells for Enhanced Antitumor Reactivity
Source: Cancers (Basel). 2022 Jun 1;14(11):2749. doi: 10.3390/cancers14112749 (PMC9179365; doi:10.3390/cancers14112749)
Supplement: Supplementary file 1 [file cancers-14-02749-s001.zip › cancers-1716133-supplementary.pdf]

Supplementary Materials: Targeting Immunosuppressive Tumor-Associated Macrophages using Innate T Cells for Enhanced Antitumor Reactivity

Yan-Ruide Li, James Brown, Yanqi Yu, Derek Lee, Kuangyi Zhou, Zachary Spencer Dunn, Ryan Hon, Matthew Wilson, Adam Kramer, Yichen Zhu, Ying Fang and Lili Yang

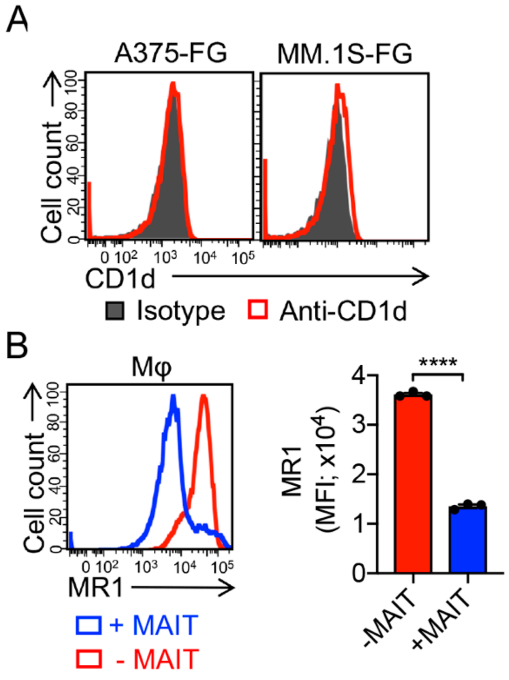

Figure S1. CD1d expression on A375 and MM.1S parental cell lines, and phenotype changes of macrophages after co-culturing with MAIT cells. Related to Figure 2 and 3. (**A**) FACS detection of CD1d expression on A375-FG and MM.1S-FG tumor cells. (**B**) FACS analysis of MR1 expression on macrophages with or without co-culturing with MAIT cells ($n = 3$). Representative of three experiments. Data are presented as the mean ± SEM. ****$p < 0.0001$, by Student's t test.

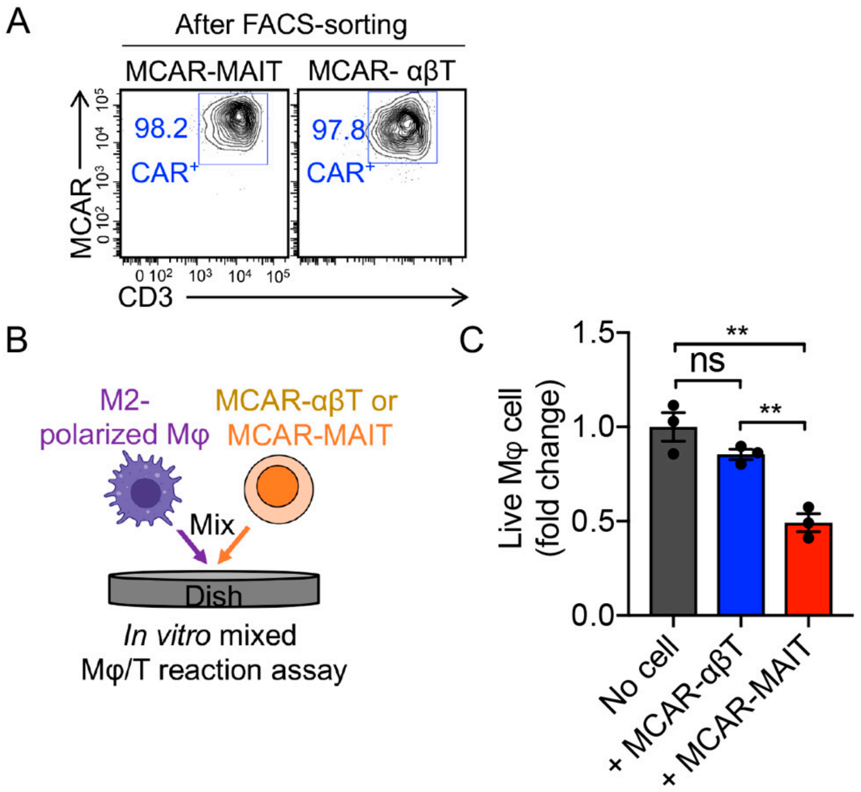

Figure S2. *In vitro* targeting immunosuppressive macrophages by MCAR-MAIT cells. Related to Figure 6. (**A**) FACS detection of MCAR expression on MCAR-αβT and MCAR-MAIT cells after FACS-sorting. (**B**) Experimental design. (**C**) FACS analysis of live macrophages 24 hours after co-culturing with MCAR-αβT or MCAR-MAIT cells. Live cells were identified as $e506^{-}CD14^{+}CD11b^{+}$ ($n = 3$). Representative of three experiments. Data are presented as the mean ± SEM. ns, not significant, $^{**}p < 0.01$, by one-way ANOVA.
